# Evaluating the Quality of Experience Performance Metric for UAV-Based Networks

**DOI:** 10.3390/s21175689

**Published:** 2021-08-24

**Authors:** Abdukodir Khakimov, Evgeny Mokrov, Dmitry Poluektov, Konstantin Samouylov, Andrey Koucheryavy

**Affiliations:** 1 Applied Mathematics & Communications Technology Institute, Peoples’ Friendship University of Russia (RUDN University), 117198 Moscow, Russia; mokrov-ev@rudn.ru (E.M.); poluektov-ds@rudn.ru (D.P.); samuylov-ke@rudn.ru (K.S.); 2 Institute of Informatics Problems, FRC CSC RAS, 119333 Moscow, Russia; 3 Department of Communication Networks and Data Transmission, The Bonch-Bruevich Saint-Petersburg State University of Telecommunications (SPBSUT), 193232 St. Petersburg, Russia; akouch@mail.ru

**Keywords:** UAV, 5G, 6G, QoE, flying network, aerial base station, emulation

## Abstract

In this work, we consider a UAV-assisted cell in a single user scenario. We consider the Quality of Experience (QoE) performance metric calculating it as a function of the packet loss ratio. In order to acquire this metric, a radio-channel emulation system was developed and tested under different conditions. The system consists of two independent blocks, separately emulating connections between the User Equipment (UE) and unmanned aerial vehicle (UAV) and between the UAV and Base station (BS). In order to estimate scenario usage constraints, an analytical model was developed. The results show that, in the described scenario, cell coverage can be enhanced with minimal impact on QoE.

## 1. Introduction

Currently, 5th generation (5G) networks are standardized and deeply researched, which allows network operators to fully implement them in their architectures. However, many “challenges” that were posed to the 5G networks were not fully resolved; therefore, the discussion of the requirements and applications of the 6th generation (6G) networks is gaining momentum in the scientific community [1,2,3]. A number of new communication scenarios are predicted, which include holographic calls, e-medicine, flying networks, and Internet of skills [4]. This in turn imposes ambitious requirements for new networks. These are ultra low latency (less than 0.1 ms), and a higher level of security and privacy, and an increase in energy efficiency. It is also assumed that a level of reliability comparable to wired networks will be provided.

To achieve such a performance, especially in emergency situations or in cases when the coverage of the terrestrial BSs is limited due to terrain conditions, the development of non-terrestrial networks (NTNs) is being considered. Such networks allow operators to provide coverage for the areas where it is impossible or economically impractical to deploy terrestrial BSs. Additionally, in cases of an emergency situation, natural disaster or simple network overload (during a concert, festival or other event), such networks would deal with the situation in the shortest possible time.

Starting with release 15, the ability to use NTNs [5] has already been added to the 3GPP standard for 5G networks, but this generation of networks still remains largely 2D oriented. In 6G networks, a fully fledged three-dimensional heterogeneous network architecture is assumed to be used. In the frame of this architecture, in addition to terrestrial BSs, Unmanned Aerial Vehicles (UAVs), High Altitude Platforms (HAPs), and satellites are to be implemented [6].

The implementation of HAPs and satellites as network elements allows for a larger coverage area, especially in difficult terrain conditions. However, they present a number of significant disadvantages, such as high transmission delay, low bandwidth and high deployment complexity. At the same time, implementing UAVs makes it possible to provide communication services at a level comparable to terrestrial BSs. This fact emphasizes that UAVs have high implementation efficiency with significantly lower complexity and deployment costs. The most relevant and realistic scenario for the NTN implementation by 2030 is the implementation of UAVs as a tool to extend the coverage of the terrestrial BSs to the areas, which are hard to cover using traditional methods.

In this paper, we first present related works, given in Section 2, where we reference several papers regarding UAV implementation in telecommunications as well as some practical trials. In Section 3, a system model is presented and several trial scenarios are considered. In Section 4, a mathematical model derives some of the constraints for the scenario usages. Section 5 describes the proposed architecture of the testbed and its technical limitations. Section 6 presents the initial data for the trial, its results and their discussion. In the conclusion, we summarize the main results and define topics for further research.

The main contributions of our paper can be summarized as follows:

- Proposed approach for a cell enhancement via UAV utilization as a repeater;
- Proposed mathematical model to estimate the system constraints;
- Developed and implement an emulator of a test network based on software defined radio (SDR) devices, which reproduces all interacting elements’ channel characteristics for the scenarios described in the system model.

The experimental results show that our proposed model allows us to enhance the cell coverage while still maintaining the required QoE levels.

## 2. Related Works

The implementation of UAVs in wireless networks for purposes of building emergency communication networks with uninterrupted network coverage and reducing the load on network sections has long attracted much attention both in scientific circles and in industry [7,8]. Due to their high mobility and deployment flexibility, UAVs are widely used in 5G networks.

The authors of [9,10] show that UAVs can be cost-effectively used as aerial platforms to provide or improve communication services of ground devices. Aerial base stations (BSs) and repeaters can be considered as examples of these platforms in cases where the terrestrial cellular network does not provide sufficient coverage. The authors of [11] examined possible use cases for drones to improve network performance, highlighting two main usage types: mobile-enabled drones (MEDs) and wireless infrastructure drones (WIDs).

In [12], to achieve maximum coverage of a UAV BS, a mathematical model calculating the optimal flight height depending on the conditions of the propagation environment is proposed.

The authors of [13], using UAVs in combination with mobile edge computing (MEC) technology with multiple access, present an algorithm for unloading traffic to UAVs, with consideration given to power consumption and movement trajectory. Expanding this approach, the authors of [14] consider offloading vehicle computations on UAVs and through UAVs to ground MEC servers, using drones as radio relays.

Application of UAVs in sensor networks and as radio bridges is also of great interest. In [15], a platform for organizing an ad hoc network using UAVs to achieve the required QoS level and GPSR and AODV is considered. Additionally, the authors of [16,17] proposed using an ad hoc network for UAVs.

In [18], a spatial network configuration scheme was constructed for additional coverage by a UAV-deployed base station to increase the QoS indicator. By dividing the coverage area into three sectors in accordance with the signal level, the authors motivate users to move towards an area with a higher signal level by offering them certain rewards. This method is partially considered in our work; however, the approach is applied in reverse, so that the UAV follows the user.

For these user-centered systems, QoE plays an essential role. The work of [19] proposes a method for optimizing this indicator through prediction, using a neural network and caching the content requested by the user on the UAV in advance. This approach, however, is effective for a network section with similar user content preferences.This paper considers an approach to the analysis of QoE in a UAV-assisted cell, studying the impact of using UAVs with directional antennas to enhance cell coverage.

To assess the effectiveness of the stochastic model of the millimeter-wave channel between a UAV and ground-based antenna, the authors of [20] carried out an analysis on an experimental setup using proprietary equipment and SDR to provide a wireless channel. A similar approach was used in [21] to build a platform for an elastic cognitive network. This approach allows one to flexibly change the network configuration by using an SDR; however, it has a high implementation cost due to the usage of proprietary equipment and the need for a special location for testing. In [22], the authors presented the possibility of emulating a wireless network using SDR or commercial radio devices in conjunction with a PC. We described a similar approach in [23] to possibly emulate signal propagation channels, potentially making changes to the signal propagation environment. This method allows an experiment to be carried out autonomously and cost-effectively, as well as its validation, before carrying out a trial using real equipment.

Table 1 presents a comparison analysis of the aforementioned works and our current paper. It can be noted that most works use either analytical or simulation approaches to derive the desired metrics; however, using an emulation approach is much closer to field trials than both of these approaches, since it emulates the channel behaviour unlike a simulation. The papers presenting experimental field trials are few in number and mostly focus on an emulation platform overview to showcase the platform’s capabilities. The current work is a continuation of [23], where the basis for the used emulation platform was to build and upgrade said emulator to analyze the QoE impact in terms of throughput in a UAV-assisted cell enhancement scenario.

## 3. System Model

We consider a scenario where the BS is insufficient to serve the user in terms of the required QoS/QoE and it implements a UAV to improve the latency and bit rate. The connection between the base station and the UAV is realized by the new radio (NR) technology using a directional antenna. The UAV is also equipped with a directional wide-angle antenna to connect to the mobile UE via NR. There are no blockers in between the BS and UAV and between the UAV and UE; thus, the free space path loss (FSPL) signal propagation model is considered. The BS always has all the information about the UAV location and the UAV always has all the information about both the BS and UE locations.

When the user is served via a UAV and moves from its preferred position, located directly under the UAV, the signal it receives from the UAV decreases. When the signal falls below a certain given threshold, the UAV starts moving towards the location where the user was at the moment the signal reached the threshold. The BS is equipped with a rotating antenna that follows UAV movements. The BS antenna coverage has a threshold area near the edges. When a UAV enters this threshold, the antenna rotates so that it is directed towards the current UAV position. The threshold depends on the antenna angular rotation speed and the distance between the BS and UAV.

**Figure 1:**
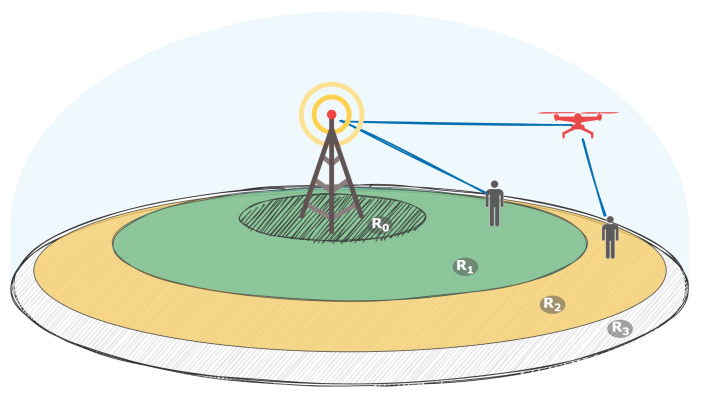
Base station and its coverage areas, divided into four areas, $R_{0}$, $R_{1}$, $R_{2}$ and $R_{3}$, corresponding to the inner, BS coverage, middle and outer areas. The inner area is restricted to UAVs since the BS antenna would not be able to catch up to them and depends on the maximum allowed UAV speed. The BS coverage area corresponds to the area where users are directly connected to the BS. The middle area is the area where UAVs can have a stable link to the BS and together with the inner area and BS coverage area completes the overall coverage of the BS directional antenna. The outer area corresponds to the BS coverage extension provided by utilizing UAVs as forwarders.

The coverage area can be divided into several areas, as depicted in Figure 1: the inner area $R_{0}$, BS coverage $R_{1}$, middle area $R_{2}$ and outer area $R_{3}$.

In the inner area, it is not advisable to use a UAV to connect to the UE, since in this area, it is possible for the BS antenna to lag behind the UAV position, resulting in connection loss. Since the area is relatively close to the BS, the UE can be connected directly to the BS with relatively good link quality. The BS coverage area is the coverage area of the multisector BS antenna, which is used to serve customers. In this area, the user can be connected via a UAV as well as directly via a multisector BS antenna. The decision regarding the connection type can be considered as an optimization problem of several parameters, such as channel quality, number of connected UEs, resource allocation scheme and others, and is not considered in the current paper. The middle area corresponds to the coverage area of the directional BS antenna, which is used for the connection between the BS and UAV. In this area, the UAV is used as a repeater to connect to the UE. Thus, UAV implementation widens the BS coverage area. The outer area is the area outside the BS coverage that can be covered by the UAV, located in the middle area. The BS is unable to provide the required signal quality directly to the user or UAV located in this area. Thus, it is advisable to utilize the UAV located in the middle area as a mediator. The outer area corresponds to the UAV coverage area under the condition that the UAV maintains its connection to the BS. In this way, the UAV essentially widens the BS coverage area even in the case of a directional antenna.

**Figure 2:**
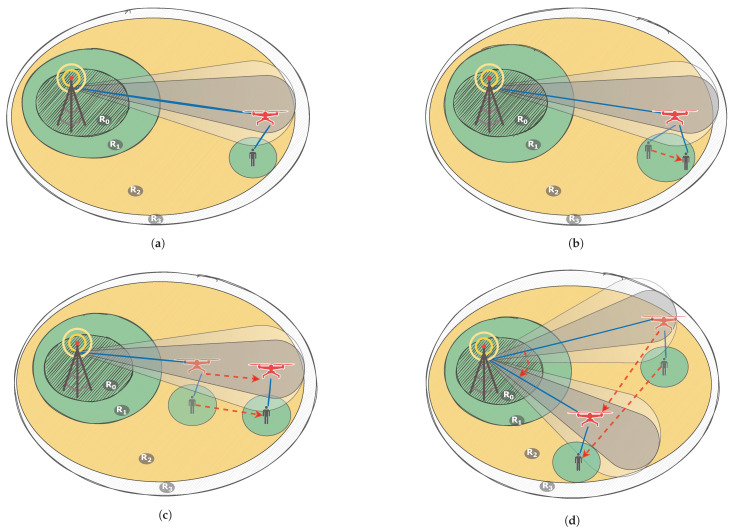
UAV utilization scenarios: (**a**) Static case, when both UAV and UE are not moving. This case is considered to prepare and test the emulator. (**b**) Semi-static case, when UE changes its position inside static UAV coverage. Used to test the UAV-UE link. (**c**) Semi-dynamic case, when UAV changes its position inside the BS directional antenna coverage and UE changes its position inside the provided UAV coverage. Used to test the BS-UAV link. (**d**) Dynamic case, when UE freely changes its position inside coverage *R*_2_ and *R*_3_ and UAV changes its position inside *R*_2_ in order to keep the link to the UE. BS directional antenna follows UAV so that the BS-UAV-UE link remains stable. This is the target test scenario.

For the described scenario we consider following cases depicted on Figure 2 to test and configure emulation test-bed.

- Case 1. This is a static case, where the BS antenna is turned towards UAV and does not change its angle. The UAV is located directly above the user and neither of them are moving. This case is proposed to test the emulator and to assure it is working as intended. This case is illustrated on Figure 2a.
- Case 2. This is a semi-static case. The BS antenna is turned towards UAV and does not change its angle. The UAV is located in its initial position and does not move. UE moves in the area where the UAV can cover it without leaving the UAV antenna coverage. This case is to ensure that the simulator/emulator correctly reacts to the user mobility. This case is illustrated on Figure 2b.
- Case 3. This is a semi-dynamic case. The BS antenna is turned towards UAV and does not change its angle. The UAV moves inside the BS antenna coverage in order to maintain user connection. The user moves in the area where the UAV can cover it without leaving the BS antenna coverage. This case tests the connection changes between BS and UAV depending on UAV position. This case is illustrated on Figure 2c.
- Case 4. This is a dynamic case. User moves in circles/spirals/given paths around the BS. The UAV moves inside the potential BS coverage in order to maintain user connection. The BS antenna is turning towards UAV when it changes its position. This scenario lets us look into the performance of the fully dynamic system. This case is illustrated on Figure 2d.

The cases were tested sequentially to ensure that the designed emulator correctly reflects the changes in signal quality resulting from the mobility of corresponding nodes. The results presented in this paper correspond to case 4, the most general case. We studied the QoE characteristic of the signal according to [24]. We considered that the transmitted traffic uses an iLBC voice codec, and, in order to properly collect the data from the emulator, the BS sent a constant stream of data to the UE. We also propose an analytical model to calculate the borders of the coverage areas.

## 4. Analytical Model

**Figure 3:**
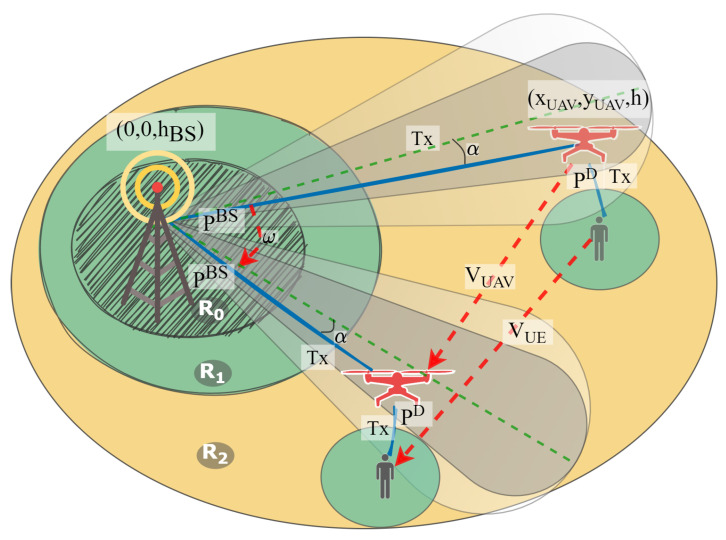
Analytical model of the considered scenario. UE moves with velocity $v_{UE}$, UAV moves with velocity $v_{UAV}$, BS directional antenna rotation speed is ω and the antenna angle is α. BS antenna is situated at altitude $h_{BS}$, UAV operates at altitude *h*, and UE is located on the ground at altitude 0. UE and UAV planar coordinates here are defined as $\left(x_{UE},y_{UE}\right)$ and $\left(x_{UAV},y_{UAV}\right)$ correspondingly. BS transmission power is $P_{tx}^{BS}$ and UAV transmission power towards UE is $p_{tx}^{D}$.

This section contains an analytical model used to calculate different constraints for the emulation. It describes ways to calculate the coverage of inner, middle and outer areas under the assumption of implementing case 4 as the most general case. The equations presented here also hold true for the other three cases described in the previous section, although in these cases they may take a more simple form. Since the UAV’s location is relatively high, we implemented an FSPL model to calculate the distance. Additionally, in this section, we do not account for the Doppler effect, since its impact on the distance calculations is neglectfully small. The proposed analytical model is illustrated by Figure 3, and all the notations are described in the current section.

BS sends signal with power $P_{tx}^{BS}$ to UAV using carrier frequency $f_{1}$ with bandwidth Δν. The UAV forwards this signal towards UE with power $P_{tx}^{UAV}$ using carrier frequency $f_{2}$ with the same bandwidth. At moment *t*, the UE is located at coordinates $\left(x_{UE},y_{UE},0\right)\left(t\right)$, the UAV is located at coordinates $\left(x_{UAV},y_{UAV},h\right)\left(t\right)$ and does not change its altitude, and the BS is always located at coordinates $\left(0,0,h_{BS}\right)$, where $h_{BS}$ is the height of BS. Let us introduce notations $SNR_{rx}^{UAV}\left(t\right)$ and $SNR_{rx}^{UE}\left(t\right)$ for the SNR at the UAV and UE at moment *t* correspondingly. The UAV changes its position as soon as $SNR_{rx}^{UE}\left(t\right)\leq SNR_{UE}^{*}$, where $SNR_{UE}^{*}$ is a threshold value of the SNR received by UE from the UAV. This means that as soon as it is unable to maintain a high enough quality, the UAV would start moving to the point closest to the UE position at time *t*. The UAV moves in such a way that its antenna is always directed towards the BS. At the same time, the BS antenna starts following the UAV position in case $SNR_{rx}^{UAV}\left(t\right)\leq SNR_{UAV}^{*}$, where $SNR_{UAV}^{*}$ is a threshold value of the SNR received by the UAV from the BS.

Since we consider the FSPL propagation model, UAV coverage can be represented as

(1) $$R_{UAV}=\sqrt{{\left(\frac{c}{4\pi f_{2}}\right)}^{2}*10^{\frac{P_{tx}^{BS}-SNR_{UE}^{*}-N}{10}}+h^{2}},$$

where *N* is the noise floor.

The BS antenna has a directive diagram f(α) and thus the SNR value on the UAV can be calculated as

(2) $$SNR_{rx}^{UAV}\left(t\right)=P_{tx}^{BS}f\left(\alpha \right)-N-{\left(\frac{4\pi d_{BS,UAV}\left(t\right)}{c}\right)}^{2},$$

where α is the angle between the antenna direction and the vector between the BS and UAS, and $d_{BS,UAV}\left(t\right)=\sqrt{(x_{UAV}^{2}+y_{UAV}^{2}+{\left(h_{BS}-h\right)}^{2}}$ is the distance between the BS and UAV.

By introducing the threshold value $SNR_{UE}^{*}$, we can calculate the coverage of the middle and outer sections as solutions for the following equations

(3) $$R_{2}=\sqrt{{\left(\frac{c}{4\pi f_{1}}\right)}^{2}*10^{\frac{P_{tx}^{BS}-SNR_{UE}^{*}-N}{10}}+{\left(h-h_{BS}\right)}^{2}},$$

(4) $$R_{3}=R_{2}+R_{UAV}.$$

Additionally, using threshold $SNR_{UAV}^{*}$, we can calculate the area where the BS antenna should start its rotation towards the drone position as a function of the UAV position

(5) $$\begin{array}{cc} G(x_{UAV},y_{UAV}): & P_{tx}^{BS}f\left(arctg\left(\frac{y_{UAV}}{x_{UAV}}\right)-\phi \right)-{\left(\frac{4\pi f_{1}}{c}\right)}^{2}\left(x_{UAV}^{2}+y_{UAV}^{2}\right)=\\ \; & SNR_{UAV}^{*}+N-{\left(\frac{4\pi f_{1}}{c}\right)}^{2}{\left(h-h_{BS}\right)}^{2}, \end{array}$$

where ϕ is the current antenna angle. Coverage of the inner area can be calculated as

(6) $$R_{0}=v_{UAV}/\omega$$

where $v_{UAV}$ is the velocity of the UAV and ω is the angular speed of the BS antenna.

By using data from this model and results of we can calculate the QoE as

(7) $$QoE=3.010e^{-4.473ploss}+1.065,$$

where ploss is packet loss—a percentage of packets lost during data transmission session between BS and UE on downlink channel in data plane. It is acquired through emulation, described in the next section.

## 5. Proposed Testbed Architecture

The main factors influencing the signal propagation channel characteristics for dynamic objects are (i) the Doppler effect, which occurs due to the non-stationarity of objects relative to one another, (ii) possible signal blockers, and (iii) changes in the state of the propagation environment. The use of the UAV as a relay base station introduces significant Doppler shift variations, which leads to an increase in the symbol delivery time and a change in the channel frequency. Scenarios with UAVs show significant Doppler variations with delay. Furthermore, the channel expansion varies in time and frequency. This is due to several factors, but the main Doppler shifts are caused by interacting transmitters and receivers in the multipath channel. Other factors determine local delays and Doppler spectral characteristics of channels, such as antenna radiation patterns and angular statistics of individual multipath propagation components (MPCs).

Most channel characteristics, such as the gain and phase shifts of individual multipath components, can be modeled by complex-static processes. However, the estimation of more MPC parameters is required for a more accurate channel model calculation.

The impulse response of the channel of moving objects, h(t,τ), is a continuous variable that depends on the signal transmission time, τ, and the signal processing time, *t*, and can be expressed as the sum of MPC:

(8) $$h\left(t,\tau \right)=\sum_{p=1}^{p}h_{p}\left(t\right)\delta \left(\tau -\tau_{p}\left(t\right)\right),$$

where *P* represents the specific MPC beam determined by the received signal strength, whereas the component $h_{p}\left(t\right)$ is defined [25] as follows:

(9) $$h_{p}\left(t\right)=\eta_{p}\hat{\eta_{p}}\left(t\right)e^{j(2\pi v_{p}\left(t\right)+\phi \left(t\right))},$$

where $\eta_{p}$ is the attenuation constant of the ray *p* and $v_{p}$ is the Doppler shift’s value on this ray.

For the time-discrete output signal r(m) calculation, it is necessary to convolve the impulse response at each time with a given sampling period *T*, i.e.,

(10) $$r\left(t\right)=\int_{\infty }^{\infty }h\left(t,t-\tau \right)s\left(\tau \right)d\left(\tau \right)=\sum_{p=1}^{p}h_{p}\left(t\right)s\left(t-\tau_{p}\right),$$

where r(t) values are calculated for t=mT values, m=1,2,….

In this paper, we analyze the impact of the proposed approach, which utilizes UAVs to expand the terrestrial network coverage while maintaining the QoE service level. To this end, we implemented an emulator of a test network based on SDR devices, which reproduces all interacting elements’ channel characteristics for the scenarios described in the system model.

The USRP 2954R radio network prototyping tool from National Instruments [26] was used for SDR devices to build the emulator. This tool has a built-in FPGA and two radio frequency channels that allow simulating networks in the range up to 6 GHz with a bandwidth of up to 160 MHz.

**Figure 4:**
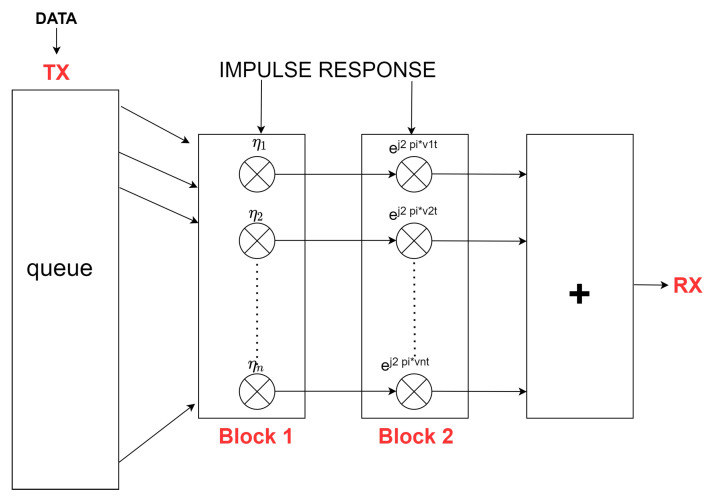
Channel emulation model. Modifying the I&Q signal by shifting the phase and amplitude of each MPC.

Since the emulator operates in real time, an FPGA module is used to perform convolution for the output signal from Equation (10), which simulates any linear time-varying channel response using simple operations, e.g., multiplication and addition. The signal pre-conversion structure specified for the FPGA is shown in Figure 4. Specifically, it is a chain of two main signal processing units between the transmitter and the receiver. These units allow modification of the characteristics of each beam at the same time following the discrete form.

The transmitter sends the generated signal s[m] represented as the in-phase and quadrature (I&Q) components of each beam (MPC). Then, the signal is divided into separate beams and enters block 1 at the separate gates for each beam and is scaled in amplitude $\hat{\eta }$ according to the left factor in Equation (11). In block 2 in Figure 4, the right factor $e^{j(2\pi v_{p}\left(t\right)+\phi \left(t\right))}$ is already added, which is responsible for phase shifts in the environment. Finally, in the last block, all beams with new characteristics are combined into an output signal r[m], which the receiver obtains in the form of I&Q.

(11) $$r\left[m\right]=\sum_{p=1}^{p}\eta_{p}e^{j(2\pi v_{p}t+\hat{\phi_{p}\left(t\right)})}.$$

In our implementation, the system operates at a bandwidth of 40 MHz, which corresponds to 200 mbps (QAM256) with a sampling time of 25 ns.

**Figure 5:**
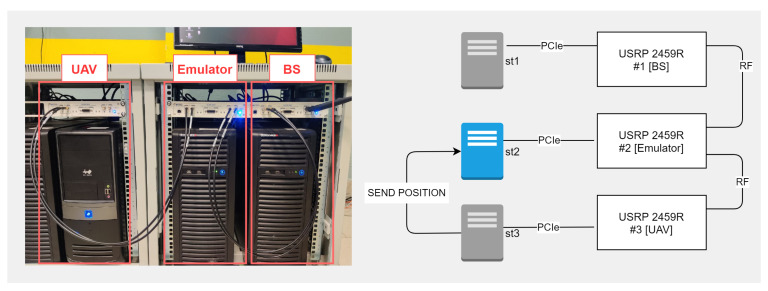
Testbed picture base station-UAV connection. (OFDMA 3300 MHz, QAM 256, bandwidth 40 MHz.)

**Figure 6:**
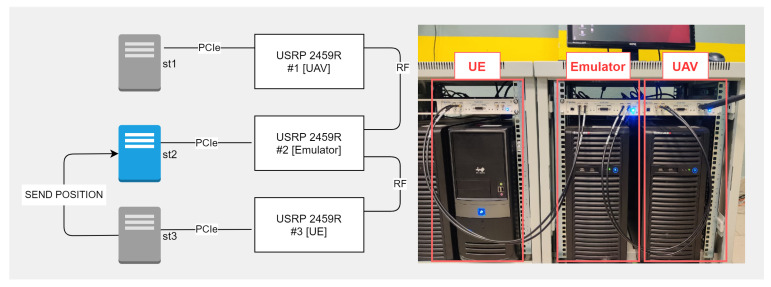
Testbed picture UAV-UE connection. (OFDMA 3500 MHz, QAM 256, bandwidth 40 MHz.)

The test setup consists of three USRP instruments in conjunction with a PC: two instruments emulate transceiver devices, and one mimics the conditions of the radio signal propagation environment. PCs obtained with a PCIe to USRP interface card are responsible for loading the compiled FPGA configuration bit files and changing SDR devices’ parameters dynamically. A scheme of the organization of element interaction is illustrated in Figure 5 and Figure 6.

The devices were connected to each other via a coaxial cable. USPR #1 was connected via the RF1 interface to RF1 USPR #2, whereas USPR #2 and #3 were connected via RF2 to RF1, respectively. When emulating data transmission, an I&Q MPC radio signal is generated at node 1 (USRP + PC) and transmitted to node 2, where the signal changes via the USRP FPGA following the specified environment parameters. The converted signal is delivered to the RF node 3 and analyzed on the PC. The reverse transmission occurs similarly. Due to the design features of the USRP, while connecting the RX and TX of two USRPs with a coaxial cable, we used signal attenuators at −30 dBi for each channel.

Each simulation object (UAV, BS, UE, signal propagation channel) is represented as an individual SDR. In view of this fact, emulation was carried out in two stages:

1. BS-UAV signal propagation channel emulation—Figure 5;
2. UAV-UE signal propagation channel emulation—Figure 6.

In order to transmit a signal from the BS to UE, the signal is transmitted to the UAV via a signal propagation channel as I&Q and the data matrix are stored at the st3 node. Then, the matrix is used in the second emulation stage, while retaining all of its properties. This method lets us minimize the number of SDRs in the testbed.

## 6. Performance Evaluation

In this section, we present the results of emulation. We consider a single user scenario, where the user moves through the network. The architecture of the network is presented in Section 3. The entire network is divided into two large areas—BS area and UAV area. The BS area is limited by a circle of radius $R_{1}$ centered at the BS, i.e., cell coverage radius. Within this area, the user is serviced by the terrestrial BS since the channel quality is sufficient to meet the QoS/QoE requirements. When the user leaves the BS area, he enters the UAV area and is transferred to the UAV. Directional antennas with rotary mechanisms are used to establish the connection between the UAV and the terrestrial BS. This makes it possible to ensure the exact direction of the signal between the transceivers regardless of the UAV location. The radius of the area in which we observe the user’s movement is limited to 2 km and corresponds to the area $R_{2}$. In view of the fact that we consider a single user scenario, the optimal location of the UAV relative to the user is strictly above him. In this case, when the user moves along the network border, it should lead to losses and, accordingly, a decrease in bandwidth and QoS/QoE, which is evaluated after the emulation is carried out. In cases when the signal is too strong, it is limited to the value of −55 dB. Table 2 presents the initial data used in the trial. The BS coverage area radius is given since usually coverage areas are known due to the cells at the deployment stage.

Since the emulator uses RSSI as the threshold, the corresponding SNR values for the analytical model are presented in the table, while RSSI values themselves are calculated.

Table 3 presents main calculated characteristics of the system, such as radius of different areas and SNR threshold values used for emulation.

**Figure 7:**
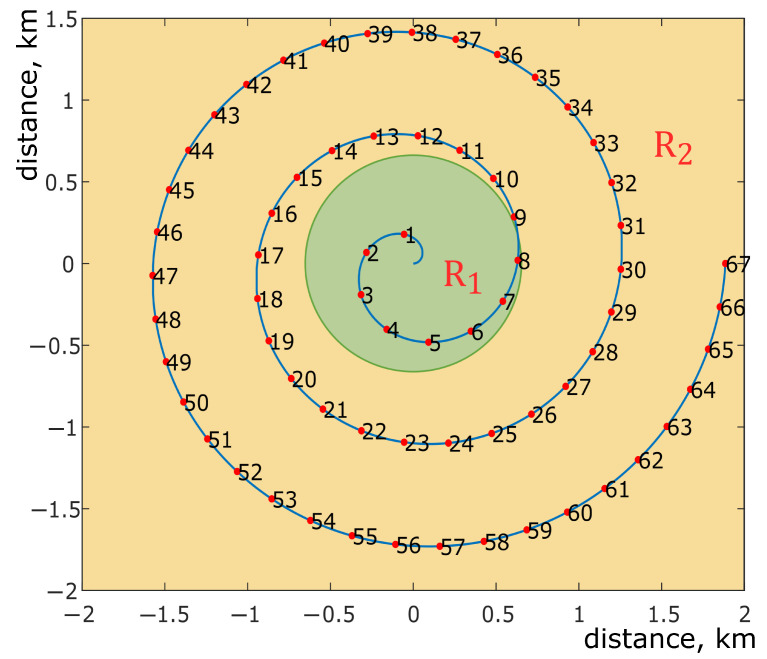
Testbed connection diagram depicts the movement trajectory of the UE with the blue line. The green circular area corresponds to the boundary between the BS coverage area, where the UE can be served by the BS directly and the middle area, where the UE should be served via the UAV. The red dots depict the measurement points at which system characteristics, such as packet loss and throughput on the UE side, were measured.

During the trial, a single user was moving through the areas along a given trajectory. When the user moves through the inner and BS coverage areas, they are directly connected to the BS; however, when they leave the BS coverage area and enter the middle area, the UE switches to the UAV located at the point where the UE trajectory leaves the BS coverage area. Figure 7 depicts the UE movement trajectory in the trial. The figure shows the BS coverage area, user trajectory and measurement points. The BS coverage area is illustrated by a green circle; the yellow area depicts the middle area. The spiral trajectory was chosen to emulate the worst case for the BS directional antenna, when it should constantly move towards the UAV that follows the user.

**Figure 8:**
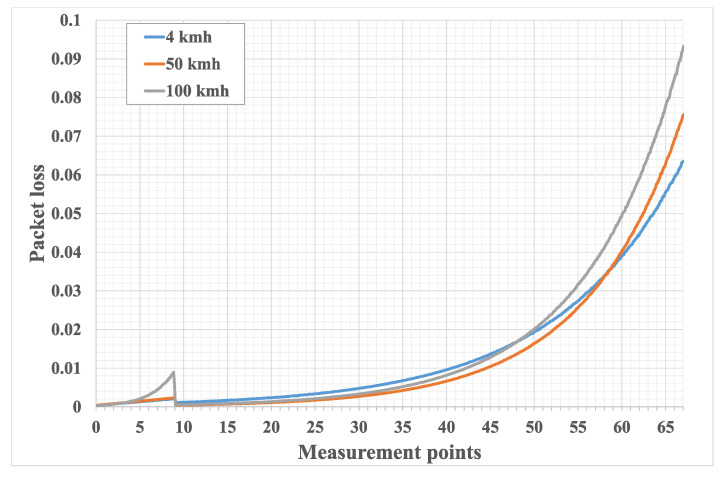
Packet loss for different UE velocities. This figure shows packet loss plotted against measurement points from Figure 7. The blue line corresponds to the pedestrian UE walking along the trajectory, the red line corresponds to the UE riding a scooter, the gray line corresponds to the UE riding a car with velocity comparable to that of the UAV.

**Figure 9:**
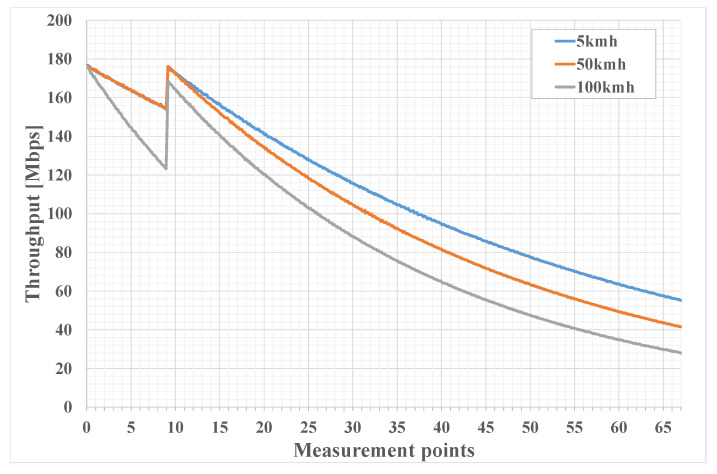
Throughput for different UE velocities. This figure shows the throughput plotted against measurement points from Figure 7. The blue line corresponds to the pedestrian UE walking along the trajectory, the red line corresponds to the UE riding a scooter, and the gray line corresponds to the UE riding a car with a velocity comparable to that of the UAV.

Figure 8 and Figure 9 show the emulation results of packet loss and throughput for different UE velocities. It can be noted that the packet loss plots slightly rise up for points 1 to 9, similarly, and throughput plots fall for these values. This is because the first nine points are located inside the inner and BS coverage areas of the cell, where UE is directly connected to the BS. Thus, a slight increase in packet loss and fall in throughput can be explained by the fact that UE distances itself from the center of the cell, moving towards its edge. A more sharp packet loss incline and throughput decline for case of UE velocity of 100 kmph is due to the fact that the BS connects to the UE via a multisector antenna and switching between sectors happens quite often for the fast moving UE with a given trajectory. Thus, more time is spent switching between sectors.It can be seen that, after leaving the green area, packet loss first falls to a lower value and then rises with distance. The initial fall is due to the fact that after the UE enters the middle area, it connects to the BS via the UAV, and since the UAV uses the directional antenna to connect to the BS, the signal actually becomes more stable and then falls as the distance grows. The same explanation holds true for throughput.

**Figure 10:**
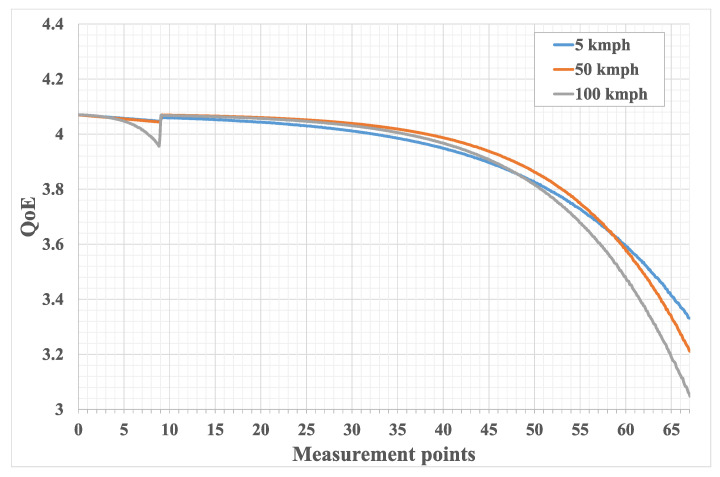
Calculation of QoE by (7) for different UE velocities.

Figure 10 presents QoE results according to the methodology of [24]. Plot behaviour is similar to that of packet loss and throughput and can be explained in a similar manner. One can see that for the considered scenario the best possible QoE value is 4.1 and it is reached inside the BS coverage area near the center of the cell. One can also note that using a UAV as a repeater to enhance the coverage keeps the QoE above 3. This means that in the considered scenario, while enhancing cell coverage from 650 m to 2 km, the overall loss in the QoE resulted in only one point at the edge of the new coverage. Even if we add an additional condition that the quality should not fall below four points, the cell coverage can still be doubled, since even for the UE moving with the same speed as the UAV, the flight speed plot falls below four points only after measurement point 30. If we consider three points as an adequate QoE, the coverage can be increased even further.

## 7. Conclusions

In order to study the performance of the UAV-assisted 5G network architecture considered in this work, a flexible functional complex testbed emulating traffic flows in such networks was developed. The deployed core network allowed us to artificially introduce delay distortions depending on the expected geographic location of the network nodes. It uses NFV in conjunction with SDR and allows virtualization of all network core functions, making it possible to quickly and flexibly change its configuration. The analytical model of the considered scenario was proposed to acquire the constraints used in the conducted emulation.

The acquired results show that using UAVs as repeaters can potentially double cell coverage area while retaining QoE level. Moreover, the coverage can be even further increased while maintaining a satisfactory QoE level with a drop of a single level.

This work scenario can be further developed by adding stationary and/or mobile blockers to a model urban environment. Another improvement would be considering multiple user scenarios while grouping users in small clusters, achieved via a single UAV. In order to prevent users from leaving their clusters, a rewards offering method can be implemented.

## Figures and Tables

**Table 1:** Selected works in comparison.

Related Works	UAV	Wireless Network Type	Metrics	Methodology	Details
[10]	✔	Unspecified	Throughput	analytical	UAV positioning optimization
[12]	✔	Cellular	Cell radius	analytical	Altitude optimization
[13]	✔	Cellular	Latency	simulation	Offloading Algorithm
[14]	✔	Unspecified	Throughput	simulation	Offloading execution time optimization
[15]	✔	Ad-hoc	Latency	simulation	QoS provisioning
[16]	✔	LoRa	Latency	simulation	Network coverage extension
[17]	✔	Ad-hoc, WiFi	Radiation pattern	experimental	The best position of the antennas
[18]	✔	Cellular	The number of served users	analytical	Spatial network configuration scheme
[19]	✔	Cellular	Transmit power	simulation	QoE optimization
[20]	✔	mmWave	Bit rate	experimental	Fading estimation
[21]	✔	Unspecified	Throughput	experimental	Code-waveform optimization
[22]	✔	Unspecified	Channel characteristics	emulation	Emulation platform overview
[23]	✕	Cellular	Channel characteristics	emulation	Emulation platform overview
This work	✔	Cellular	Throughput	emulation, analytical	Emulation platform, QoE optimization

**Table 2:** Initial data.

Notation	Value	Description
Δν	40 MHz	bandwidth
$f_{1}$	3300 MHz	BS-UAV frequency
*c*	3 × 10${}^{8}$ m/s	light speed
	QAM 256	modulation
$P_{BS}$	21 dBm	transmission power of BS
$P_{UAV}$	21 dbm	transmission power of UAV towards BS
$\phi_{BS}$	15	BS-UAV antenna angle
$\phi_{UAV,BS}$	15	UAV-BS antenna angle
$\phi_{UAV,UE}$	90	UAV-UE antenna angle
f(x)	cos(x)	Antenna direction diagram
ω	1 rad/s	BS an a rotation speed
*N*	−100 dB	noise floor
$h_{BS}$	20 m	height of BS
*h*	55 m	UAV flight altitude
$SNR_{UE}^{*}$	40 dbm	threshold SNR between UAV and UE
$SNR_{UAV}^{*}$	12 dbm	threshold SNR between BS and UAV
$f_{2}$	3500 Mhz	UAV-UE frequency
$v_{UAV}$	100 km/h	UAV flight speed
$v_{1}$	4 km/h	pedestrian UE velocity
$v_{2}$	50 km/h	bus UE velocity
$v_{3}$	100 km/h	car UE velocity
$R_{1}$	650 m	BS coverage radius

**Table 3:** Calculated data.

Notation	Value	Description
$R_{0}$	27 m	inner area radius
$R_{2}$	2038 m	middle area radius
$R_{UAV}$	54 m	UAV coverage area
$RSSI_{UE}^{*}$	−60 dbm	threshold RSSI between UAV and UE
$RSSI_{UAV}^{*}$	−88 dbm	threshold RSSI between BS and UAV
